# A real-world pharmacovigilance study of drug-induced QT interval prolongation: analysis of spontaneous reports submitted to FAERS

**DOI:** 10.3389/fcvm.2024.1363382

**Published:** 2024-05-13

**Authors:** Haowen Tan, Xida Yan, Ying Chen, Guili Huang, Luping Luo, Wenjun Li, Weiwei Lan, Cheng Chen, Xin Xi

**Affiliations:** ^1^ Department of Pharmacy, Wuzhou Red Cross Hospital, Wuzhou, Guangxi, China; ^2^ Office of Good Clinical Practice, Wuzhou Red Cross Hospital, Wuzhou, Guangxi, China; ^3^ Department of Pharmacy, Mianyang Central Hospital, Mianyang, Sichuan, China; ^4^ Department of Pharmacy, The Third Affiliated Hospital of Chongqing Medical University, Chongqing, China

**Keywords:** QT interval prolongation, pharmacovigilance, FAERS, data mining, reporting odds ratio

## Abstract

**Purpose:**

To identify the most commonly reported drugs associated with QT interval prolongation in the FDA Adverse Event Reporting System (FAERS) and evaluate their risk for QT interval prolongation.

**Methods:**

We employed the preferred term (PT) “electrocardiogram QT prolonged” from the Medical Dictionary for Regulatory Activities (MedDRA) 26.0 to identify adverse drug events (ADEs) of QT interval prolongation in the FAERS database from the period 2004–2022. Reporting odds ratio (ROR) was performed to quantify the signals of ADEs.

**Results:**

We listed the top 40 drugs that caused QT interval prolongation. Among them, the 3 drugs with the highest number of cases were quetiapine (1,151 cases, ROR = 7.62), olanzapine (754 cases, ROR = 7.92), and citalopram (720 cases, ROR = 13.63). The two most frequently reported first-level Anatomical Therapeutic Chemical (ATC) groups were the drugs for the nervous system (*n* = 19, 47.50%) and antiinfectives for systemic use (*n* = 7, 17.50%). Patients with missing gender (*n* = 3,482, 23.68%) aside, there were more females (7,536, 51.24%) than males (5,158, 35.07%) were involved. 3,720 patients (25.29%) suffered serious clinical outcomes resulting in deaths or life-threatening conditions. Overall, most drugs that caused QT interval prolongation had early failure types according to the assessment of the Weibull's shape parameter (WSP) analysis.

**Conclusions:**

Our study offered a list of drugs that frequently caused QT interval prolongation based on the FAERS system, along with a description of some risk profiles for QT interval prolongation brought on by these drugs. When prescribing these drugs in clinical practice, we should closely monitor the occurrence of ADE for QT interval prolongation.

## 1 Introduction

The QT interval, measured as the interval between the initiation of the Q wave and the termination of the T wave on an electrocardiogram, is a measure of ventricular depolarisation and repolarisation (1). As per the current diagnostic criteria, a corrected QT (QTc) interval longer than 450 milliseconds (ms) for men and 470 ms for women is considered a “prolonged QTc interval” (2). QT interval prolongation is correlated with the risk of cardiac events (3), and one of the most severe consequences of a prolonged QT interval is a ventricular arrhythmia known as torsade de pointes (TdP), which can lead to sudden death (4, 5).

In general, hypocalcemia, hypokalemia, coronary artery disease, hypertension, diabetes mellitus, medications, and other factors result in QT interval prolongation (6–11). According to reports, antiarrhythmic medications, macrolides, fluoroquinolones, antifungal medications, antipsychotic medications, antihistamines, antiviral medications, anticancer medications, diuretics, and others might cause QT interval prolongation (12–14). Considering the medication's safety, drug-induced QT interval prolongation has been an important factor behind the removal or restricted use of medications during the past 20 years.

Because of the unpredictable and dangerous outcomes, drug-induced QT interval prolongation is still a concern that needs to be continuously monitored during treatment. Currently, there have been few studies that attempt to use big data mining in real-world pharmacovigilance to monitor drug-induced QT interval prolongation. Food and Drug Administration (FDA) Adverse Event Reporting System (FAERS) is a spontaneous reporting system that gathers a large number of adverse drug event (ADE) reports. The purpose of this database is to assist the FDA's post-marketing safety surveillance program for pharmaceutical and therapeutic biologic products. It can also be used to display adverse event profiles from actual clinical settings. Utilizing the FAERS database, we conducted data mining on ADEs with drug-induced QT interval prolongation in order to provide prescribers with reference information to ensure drug safety, as well as to give valuable information for pharmacoepidemiology.

## 2 Methods

### 2.1 Data source and mining

We obtained the American standard code for information interchange (ASCII) data file in the FAERS database (https://fis.fda.gov/extensions/FPD-QDE-FAERS/FPD-QDE-FAERS.html) from 2004 to 2022. The ASCII data file gathered seven subfiles individually, including DEMOyyQq (demographic information), DRUGyyQq (drug information), REACyyQq (adverse event), OUTCyyQq (outcome information), RPSRyyQq (report source), THERyyQq (treatment data), and INDIyyQq (indication for drug).

SAS (9.4) was applied in our study for data mining. We chose PRIMARYID, CASEID, and FDA_DT in the DEMO table and sorted them in the order of CASEID, FDA_DT, and PRIMARYID in accordance with the FDA's method for removing duplicate reports. We maintained the maximum value of FDA_DT for reports that had the same CASEID. Then, we reserved the report with the highest value of PRIMARYID for reports with the same CASEID and FDA_DT. The primary suspect drug (PS) was found by applying the preferred term (PT) “Electrocardiogram QT Prolonged” with the PT code 10014387 from the Medical Dictionary for Regulatory Activities (MedDRA) 26.0.

### 2.2 Discrimination of objective drugs

There was a lack of standardization in filling in the drug names in the FAERS database because of the wide range of reporting groups, including both healthcare professionals like physicians or pharmacists, and non-healthcare professionals like consumers and lawyers. For the same drug, we gathered all forms of drug names reported in FAERS, including generic name, brand name, drug code, active pharmaceutical ingredients, and their corresponding non-standard names in FAERS, in order to increase the data's accuracy. Then we categorized every drug using the World Health Organization's Anatomical Therapeutic Chemical (ATC) classification (https://www.whocc.no/atc_ddd_index/). Afterwards, we reviewed the package inserts of these drugs to see if there was an ADE for QT interval prolongation in their labels.

### 2.3 Disproportionality analysis

To find the ADE signal, we applied the reporting odds ratio (ROR) of the disproportionality method. This method compares the proportion of targeted events of the targeted drugs with the proportion of targeted events of all other drugs in order to mine potential risk signals of ADEs (15). The RORs for the drugs that caused QT interval prolongation were calculated by using a two-by-two contingency table, as shown in Supplementary Table S1. ROR=(a/c)(b/d)=ad/bc, and 95% confidence interval $(CI)=e^{\ln ROR\pm \sqrt{\frac{1}{a}+\frac{1}{b}+\frac{1}{c}+\frac{1}{d}}}$. When a ≥ 3 and the lower end of the 95% CI for the ROR value is higher than 1, the potential risk signal of ADE is satisfied. Respectively, we used the ASCII data file encompassing the time from the FDA approval time of each drug to the fourth quarter of 2022 in order to increase the accuracy of the computation for the ROR of each drug.

### 2.4 Time-to-onset analysis

Time-to-onset (TTO) was outlined as the period of time between the ADE occurrence date (EVENT_DT in the DEMO file) and the beginning date of drug use (START_DT in the THER file). In the meantime, we removed any inaccurate date inputs, missing specific data, or input errors (EVENT_DT comes before START_DT). Additionally, the TTO was assessed by using the medians, interquartile ranges (IQR), and Weibull's shape parameter (WSP) (16). The shape of the Weibull distribution was described by two parameters: scale (α) and shape (β). Early failure types are characterized by an ADE hazard that decreases with time (β <1% and 95% CI <1); random failure types are characterized by an ADE hazard that occurs continuously over time (β is equal to or close to 1 and its 95% CI contains the value 1); and wearout failure types are characterized by an increase in ADE hazard with time (β >1% and 95% CI >1) (17).

## 3 Results

### 3.1 Descriptive analysis

**Figure 1:**
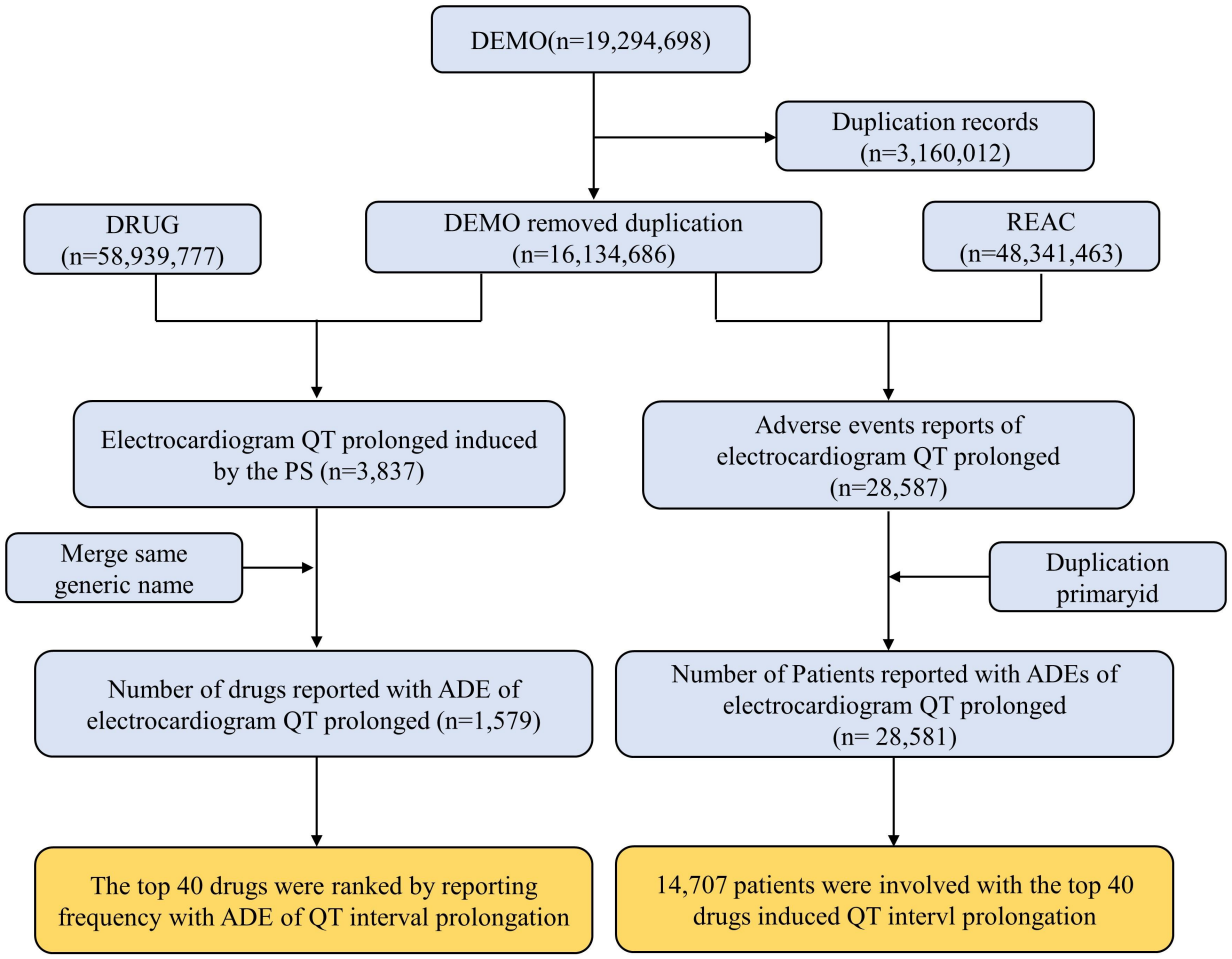
Flow chart for identification cases of QT interval prolongation in our study. PS, primary suspect drug; ADE, adverse drug event.

**Figure 2:**
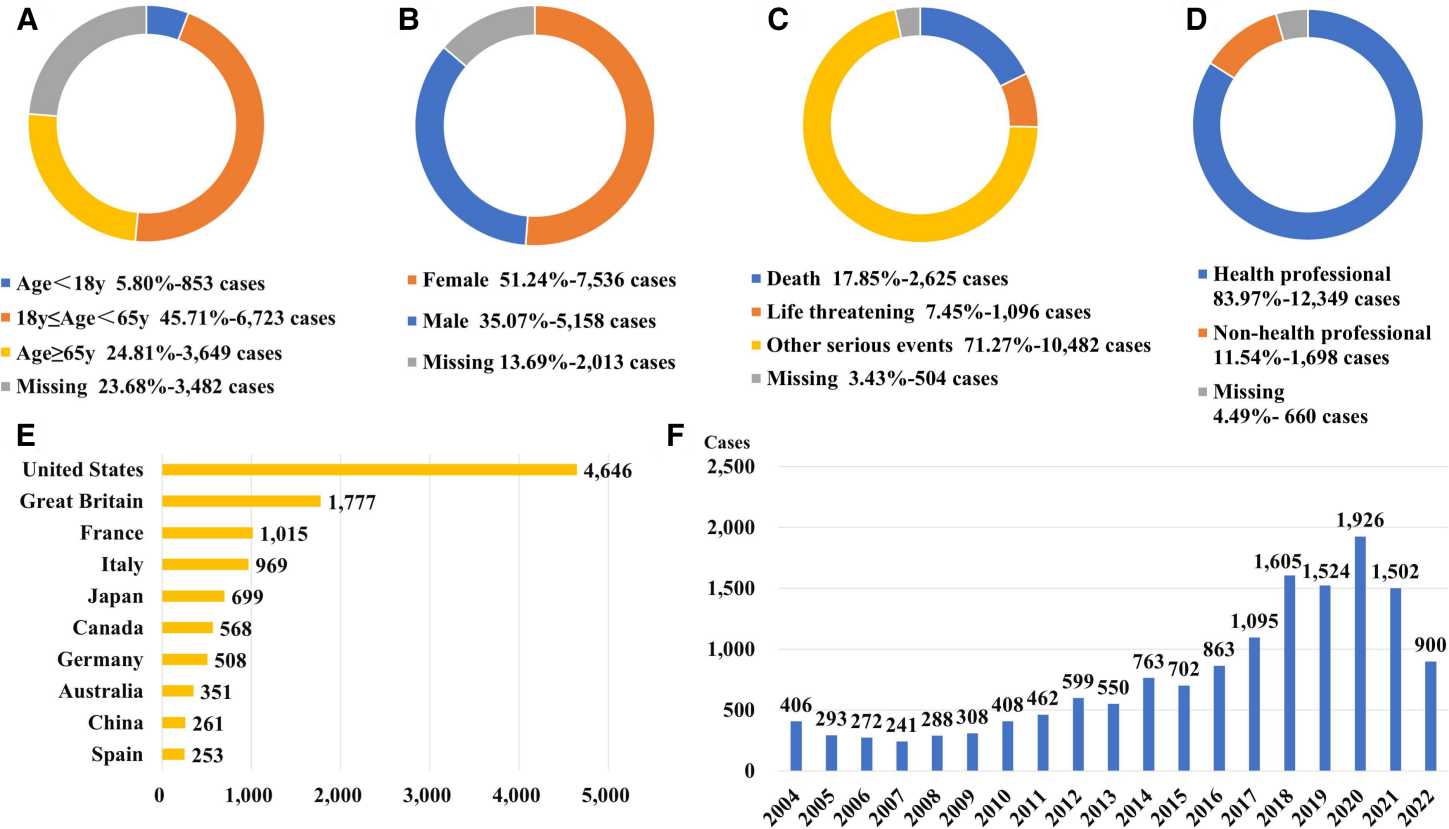
Patients’ characteristics and reporting information for the top 40 drugs with ADE of QT interval prolongation. (**A**) Distribution of patients’ age (**B**) Distribution of patients’ gender. (**C**) Distribution of patients’ outcome (**D**) Distribution of the reporters (**E**) Top 10 countries with the most sources of reports. (**F**) Distribution of the reporting year.

Following data mining, we discovered 1,579 different drugs responsible for 28,581 patients associated with “electrocardiogram QT prolongation”. The top 40 drugs-induced QT interval prolongation cumulatively involved 14,707 patients. The process of data mining is shown in Figure 1. Among 14,707 patients, there were 6,723 patients aged 18–65 years (45.71%). Patients with missing gender (*n* = 3,482, 23.68%) aside, there were more female patients (*n* = 7,536, 51.24%) than male patients (*n* = 5,158, 35.07%). In total, 3,721 patients (25.30%) experienced death or life-threatening clinical outcomes. Additionally, the United States reported the most number of cases (*n* = 4,646) out of all the reporting nations. When compared to non-healthcare professionals, healthcare professionals reported more cases. There has been an upward trend in the reporting of ADEs for QT interval prolongation since 2004, which should be considered of sufficient concern. More details on patients' characteristics and reporting information for the top 40 drugs are shown in Figure 2 and Supplementary Table S2.

### 3.2 Culprit-drug list induced QT interval prolongation

**Figure 3:**
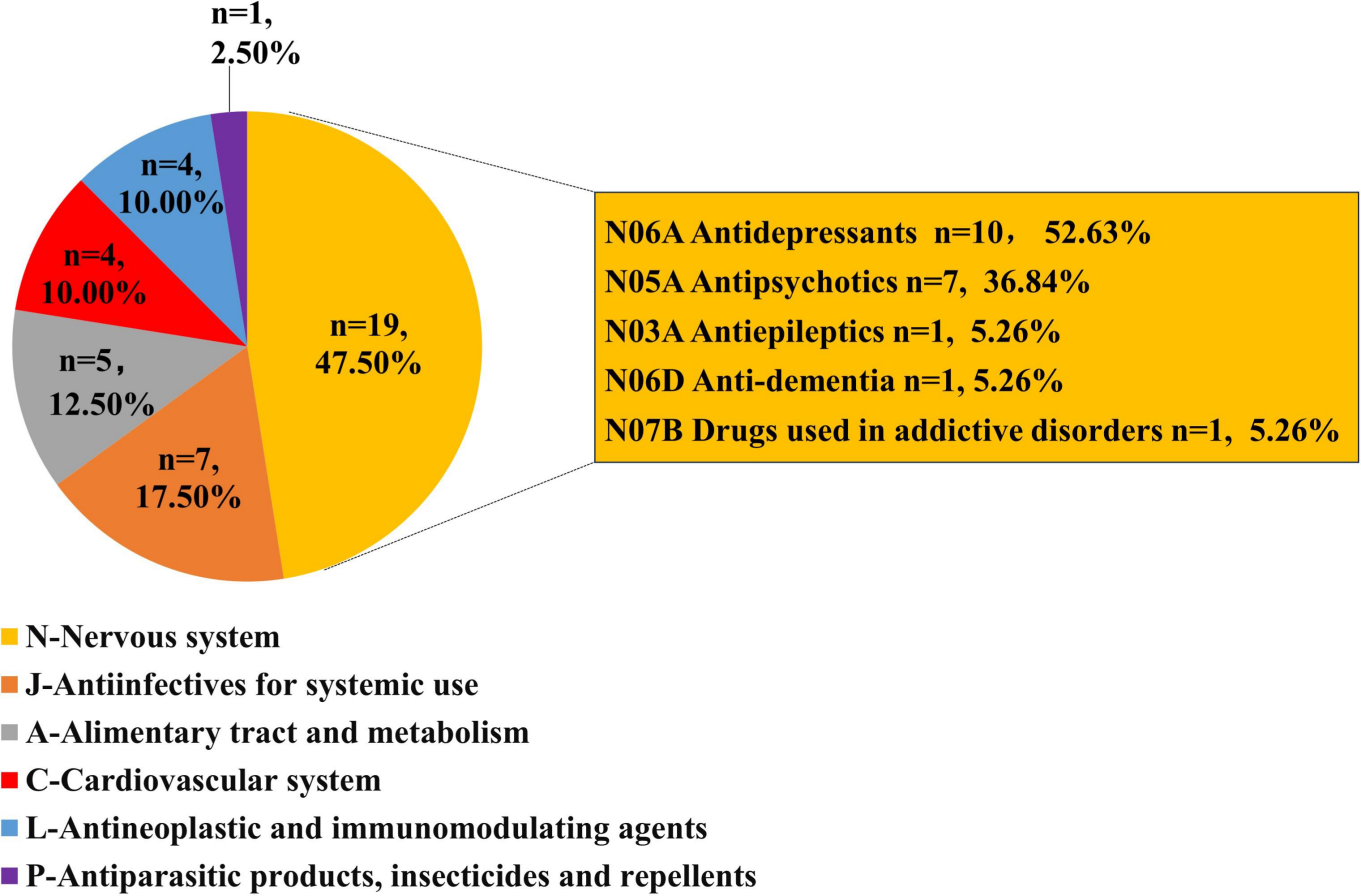
Classification of top 40 drugs associated with ADE of QT interval prolongation.

The top 40 drugs (Table 1) by overall frequency of presence, with cases reported ranging from 155 to 1,151 and RORs ranging from 1.19 to 312.67, were taken into further analysis. The top 3 drugs with the highest number of reported cases were quetiapine (1,151 cases), olanzapine (754 cases), and citalopram (720 cases), and the 3 drugs with the highest ROR values were cisapride (ROR = 312.67, 95% CI: 281.35–347.47), bedaquiline (ROR = 125.94, 95% CI: 109.23–145.21), and dofetilide (ROR = 37.53, 95% CI: 33.79–41.70). According to the first level of the ATC classification, we divided the 40 drugs into categories. As shown in Figure 3, the most common drugs causing QT interval prolongation in the FAERS database were the drugs for the nervous system (ATC N, *n* = 19, 47.50%) and antiinfectives for systemic use (ATC J, *n* = 7, 17.50%). When we further classified the drugs for the nervous system at the third level of the ATC classification, we discovered that antidepressants (ATC N06A, *n* = 9, 47.37%) and antipsychotics (ATC N05A, *n* = 7, 36.84%) were the two primary drug classes.

**Table 1:** The top 40 drugs associated with ADE of QT interval prolongation.

Ranking	ATC code	Drug	Cases	ROR (95% Cl)
1	N05AH04	Quetiapine	1,151	7.62 (7.18–8.08)
2	N05AH03	Olanzapine	754	7.92 (7.36–8.51)
3	N06AB04	Citalopram	720	13.63 (12.65–14.68)
4	L01EA03	Nilotinib	688	14.00 (12.97–15.10)
5	N05AH02	Clozapine	609	4.87 (4.50–5.28)
6	C01BD01	Amiodarone	540	13.27 (12.18–14.46)
7	P01BA02	Hydroxychloroquine	511	16.56 (15.16–18.08)
8	N06AX12	Bupropion^a^	489	7.18 (6.57–7.86)
9	N06AB10	Escitalopram	483	11.36 (10.38–12.44)
10	J01FA10	Azithromycin	481	13.64 (12.46–14.93)
11	A04AA01	Ondansetron	430	12.08 (10.98–13.29)
12	A03FA02	Cisapride	413	312.67 (281.35–347.47)
13	A07DA03	Loperamide	397	17.99 (16.28–19.87)
14	N06AX16	Venlafaxine	384	3.80 (3.44–4.20)
15	N07BC02	Methadone	374	27.58 (24.89–30.57)
16	C01BD04	Dofetilide	359	37.53 (33.79–41.70)
17	C03CA01	Furosemide^a^	350	8.86 (7.97–9.85)
18	N06AB06	Sertraline	342	3.45 (3.10–3.84)
19	L04AA27	Fingolimod	341	2.17 (1.95–2.41)
20	N05AX12	Aripiprazole	339	3.01 (2.70–3.35)
21	N05AX08	Risperidone	317	3.13 (2.80–3.50)
22	N06AB03	Fluoxetine	308	7.85 (7.01–8.78)
23	N05AE04	Ziprasidone	273	15.89 (14.10–17.91)
24	J01MA14	Moxifloxacin	273	7.73 (6.86–8.71)
25	N06DA02	Donepezil	269	19.23 (17.04–21.70)
26	N06AX11	Mirtazapine	255	7.27 (6.43–8.23)
27	N05AD01	Haloperidol	253	12.06 (10.65–13.65)
28	L01EF02	Ribociclib	246	8.93 (7.87–10.13)
29	J01MA12	Levofloxacin	246	3.45 (3.04–3.91)
30	N06AB05	Paroxetine	230	2.40 (2.11–2.73)
31	C08CA01	Amlodipine^a^	227	1.97 (1.72–2.24)
32	J01MA02	Ciprofloxacin	210	2.18 (1.90–2.50)
33	A02BC01	Omeprazole^a^	208	2.89 (2.52–3.31)
34	J04AK05	Bedaquiline	205	125.94 (109.23–145.21)
35	J01FA09	Clarithromycin	194	6.51 (5.65–7.50)
36	L02BG04	Letrozole^a^	193	5.10 (4.43–5.88)
37	N06AX21	Duloxetine	175	1.19 (1.03–1.38)
38	N03AX09	Lamotrigine^a^	159	1.53 (1.31–1.79)
39	J02AC01	Fluconazole	156	9.60 (8.20–11.24)
40	A10BA02	Metformin^a^	155	1.35 (1.15–1.58)

ADE, adverse drug event; CI, confidence interval; ROR, reporting odds ratio.

^a^ Adverse events of QT interval prolongation that appears in the drug label.

### 3.3 Time-to-onset analysis

In order to guarantee the accuracy of the TTO analysis, we excluded any data that was incorrect or missing. As a result, fewer cases were included for further analysis than were actually reported. With the exception of ondansetron, all shape parameters' 95% CI upper limits were smaller than 1, indicating that these medications have early failure types, according to the assessment of the WSP analysis. Due to its shape parameter of 1.04 (95% CI: 0.86–1.23), ondansetron had a random failure type. In Table 2, the results of TTO and WSP analyses for the top 40 drugs linked to reports of QT interval prolongation are presented.

**Table 2:** The TTO for the top 40 drugs associated with ADE of QT interval prolongation.

SN	Drug	Cases	TTO (days)		Webibulll distribution				Failure type
					Scale parameter		Shape parameter		
		*n*	Median	IQR	α	95% CI	β	95% CI	
1	Quetiapine	314	1.00	1.00–2.00	19.65	13.54–25.77	0.38	0.35–0.41	Early Failure
2	Olanzapine	225	8.00	1.00–100.00	48.73	30.89–66.58	0.38	0.34–0.42	Early Failure
3	Citalopram	185	20.00	1.00–555.00	119.23	69.17–169.30	0.36	0.32–0.40	Early Failure
4	Nilotinib	266	18.00	6.75–110.75	64.55	48.56–80.54	0.51	0.47–0.56	Early Failure
5	Clozapine	232	826.00	25.50–2696.25	987.41	717.70–1257.13	0.49	0.44–0.55	Early Failure
6	Amiodarone	138	11.50	3.00–103.00	64.14	39.19–89.10	0.45	0.40–0.51	Early Failure
7	Hydroxychloroquine	46	3.00	1.00–6.25	16.46	0.72–32.19	0.32	0.26–0.38	Early Failure
8	Bupropion	12	2.00	1.00–166.00	56.85	−51.61–165.32	0.31	0.18–0.45	Early Failure
9	Escitalopram	137	7.00	1.00–67.00	31.05	17.85–44.24	0.42	0.37–0.47	Early Failure
10	Azithromycin	151	2.00	1.00–6.00	5.30	3.94–6.66	0.66	0.59–0.73	Early Failure
11	Ondansetron	62	1.00	1.00–4.00	2.85	2.13–3.57	1.04	0.86–1.23	Random Failure
12	Cisapride	159	353.00	18.00–1096.00	538.43	364.04–712.83	0.50	0.44–0.57	Early Failure
13	Loperamide	19	8.00	1.00–1833.00	171.55	−73.05–416.14	0.33	0.21–0.46	Early Failure
14	Venlafaxine	83	1.00	1.00–267.00	42.17	16.63–67.70	0.38	0.32–0.44	Early Failure
15	Methadone	51	25.00	3.00–517.00	130.34	32.50–228.17	0.39	0.30–0.47	Early Failure
16	Dofetilide	147	2.00	1.00–23.00	21.36	11.07–31.65	0.36	0.32–0.40	Early Failure
17	Furosemide	61	2.00	2.00–258.00	45.12	14.71–75.53	0.40	0.32–0.47	Early Failure
18	Sertraline	101	92.00	1.00–530.50	205.18	104.09–306.27	0.42	0.35–0.48	Early Failure
19	Fingolimod	281	1.00	1.00–1.00	3.12	2.25–4,00	0.45	0.41–0.48	Early Failure
20	Aripiprazole	79	10.00	1.00–56.00	36.12	16.14–56.10	0.42	0.36–0.49	Early Failure
21	Risperidone	54	1.00	1.00–8.75	13.90	3.17–24.63	0.37	0.33–0.44	Early Failure
22	Fluoxetine	45	2.00	1.00–157.00	49.04	5.35–93.73	0.35	0.27–0.42	Early Failure
23	Ziprasidone	66	29.50	1.00–92.25	51.53	24.23–78.84	0.48	0.39–0.57	Early Failure
24	Moxifloxacin	114	1.00	1.00–4.00	6.14	3.50–8.77	0.46	0.40–0.51	Early Failure
25	Donepezil	55	118.00	14.00–539.00	220.91	111.53–330.29	0.56	0.44–0.68	Early Failure
26	Mirtazapine	117	1.00	1.00–7.00	7.49	3.97–11.00	0.41	0.36–0.46	Early Failure
27	Haloperidol	51	1.00	1.00–3.00	6.09	1.84–10.35	0.42	0.35–0.49	Early Failure
28	Ribociclib	79	15.00	9.00–31.00	34.43	22.11–46.75	0.65	0.55–0.76	Early Failure
29	Levofloxacin	96	2.00	1.00–6.00	7.43	4.11–10.75	0.48	0.42–0.54	Early Failure
30	Paroxetine	57	111.00	1.00–453.00	129.07	43.80–214.33	0.42	0.33–0.50	Early Failure
31	Amlodipine	30	28.00	1.00–937.00	160.44	8.47–312.41	0.40	0.28–0.52	Early Failure
32	Ciprofloxacin	60	3.00	1.00–7.75	7.97	4.19–11.76	0.57	0.47–0.66	Early Failure
33	Omeprazole	20	2.00	1.00–170.00	25.48	−6.12–57.09	0.38	0.25–0.50	Early Failure
34	Bedaquiline	136	26.00	13.00–76.75	46.46	36.93–55.99	0.87	0.76–0.98	Early Failure
35	Clarithromycin	72	3.00	2.00–9.75	6.52	4.57–8.48	0.82	0.69–0.94	Early Failure
36	Letrozole	105	16.00	12.00–44.00	45.38	30.39–60.37	0.62	0.53–0.70	Early Failure
37	Duloxetine	38	21.50	1.00–211.00	67.82	13.83–121.82	0.42	0.32–0.53	Early Failure
38	Lamotrigine	29	1.00	1.00–100.50	28.10	3.82–52.39	0.45	0.32–0.57	Early Failure
39	Fluconazole	38	8.00	1.75–17.50	24.47	4.96–43.97	0.42	0.33–0.52	Early Failure
40	Metformin	23	1.00	1.00–180.00	25.75	−6.97–58.47	0.34	0.24–0.45	Early Failure

ADE, Adverse drug event; ATC, Anatomical Therapeutic Chemical; CI, Confidence interval; IQR, interquartile ranges; SN, serial number; TTO, time-to-onset.

## 4 Discussion

The standardized code of ADE terms is the key to identifying signals of drug safety in millions of ADEs. MedDRA is a clinically validated international medical terminology used to classify ADEs. As an important signal recognition tool, it has been increasingly used for drug safety analysis. The PT names associated with QT interval prolongation recorded in the FAERS are “electrocardiogram QT prolonged” and “long QT syndrome”. Long QT syndrome has been classified as being either congenital or acquired (18). However, congenital QT prolongation is caused by mendelian genetic disorders (19), and it is not associated with our current study. In order to improve the accuracy of our study, we only used the PT name “electrocardiogram QT prolonged” when we assessed the drug-induced prolongation of the QT interval in the FAERS database.

In our study, we found that drug-induced QT prolongation occurred in a higher percentage of females than males, which was consistent with previous studies (20, 21). The 20 ms longer baseline QTc intervals in women compared to men had been considered to be linked to the increased risks in women (22). It was reported that testosterone accelerates ventricular repolarization (23). Furthermore, the expression of several repolarizing ion-channels, including human ether-a-go-go-related gene (hERG), was found to be decreased in female hearts than in male hearts (24). Multiple factors make women more likely to be induced by drugs to prolong the QT interval. Rosen et al. (12) reported that age above 65 was a high-risk factor for QT interval prolongation. In contrast to previous studies, our study found that the proportion of QT interval prolongation in the 18–64 age group was higher than in the over-65 age group. This may be closely associated with the fact that the top 40 drugs for QT interval prolongation reported in the FAERS database included a higher percentage of patients (26.59%) who were 18–65 years old and took antidepressants and antipsychotics. Based on epidemiological investigations, mental disease tends to start at a younger age (25, 26).

Over the past decade, the effects on the QT interval and the associated risk of heart rhythm disorders of antidepressants had been raising concerns, while the risks of QT interval prolongation varied with different antidepressants. Among the most common antidepressants that caused QT interval prolongation in our study, citalopram (ROR = 13.63, 95% CI: 12.65–14.68) and escitalopram (ROR = 11.36, 95% CI: 10.38–12.44) had a higher risk of QT interval prolongation, and venlafaxine (ROR = 3.80, 95% CI: 3.44–4.20), sertraline (ROR = 3.45, 95% CI: 3.10–3.44), paroxetine (ROR = 2.40, 95% CI: 2.11–2.73), and duloxetine (ROR = 1.19, 95% CI: 1.03–1.38) had a relatively low risk of QT interval prolongation, similar to the findings of Funk et al. (27) and Jasiak et al. (28). Consequently, it is critical to appropriately evaluate the risk of medicine when treating depression, and we advise selecting antidepressants with a reduced risk for individuals who are at a high risk of developing QT interval prolongation.

In clinical practice, reports of QT interval prolongation caused by antipsychotics are more frequent. Currently, except for lurasidone, cariprazine, and brexpiprazole, most antipsychotic drugs can cause QT interval prolongation at therapeutic doses or in overdose by blocking the kalium current (IKr) ion channels (29). Ziprasidone, quetiapine, and risperidone showed the most significant inhibitory effects on IKr (30). Additionally, the cytochrome P450 (CYP) enzyme is responsible for the majority of antipsychotic drug metabolism, so combining them with other medications may increase the risk of QT interval prolongation. In our study, quetiapine (1,151 cases), olanzapine (754 cases), and clozapine (609 cases) were three of the top five drugs causing QT interval prolongation. These drugs belong to the second generation of antipsychotics, which gained popularity because they caused fewer side effects than those from the first generation. Since they were often utilized in clinics, more patients were observed to experience QT interval prolongation as a result.

Systemic anti-infective drugs, of which macrolides and fluoroquinolones were the most common, were another class of drugs that frequently resulted in QT interval prolongation in our study. The mechanism for the QT interval prolongation induced by macrolides is the inhibition of rectification of IKr ion channels and the suppression of auto-metabolism by CYP3A4 enzyme (31, 32). Fluoroquinolones, including ciprofloxacin, levofloxacin, and moxifloxacin, have been reported to cause QT interval prolongation and TdP (33–36). In addition, azole antifungal drugs are mostly potent inhibitors of the CYP3A4 enzyme, and when combined, they can increase the concentration of other drugs that prolong the QT interval. We are unable to avoid utilizing macrolides, fluoroquinolones, and azole antifungals since they are essential in the treatment of infectious illnesses. Therefore, we must be alert to the potential side effect of QT interval prolongation when using these drugs and avoid concomitantly using drugs that may interact with them.

In addition, our study discovered that the adverse effect of QT interval prolongation was not mentioned in the drug package inserts for seven medications, including bupropion, furosemide, amlodipine, omeprazole, letrozole, lamotrigine, and metformin. For these drugs, QT interval prolongation is their new risk signal for ADE. A list of drugs from CredibleMeds (available at www.crediblemeds.org) that increase the risk of QT interval prolongation and TdP includes furosemide and omeprazole in the conditional risk of TdP. This shows that omeprazole and furosemide might prolong the QT interval in specific situations, which may result in TdP. It is worth noting that on March 31, 2021, FDA released information that lamotrigine may increase the risk of arrhythmia in patients with heart disease. However, studies found that lamotrigine didn't prolong the QT interval in healthy subjects (37). Therefore, we need to pay attention to the risk of QT interval prolongation caused by lamotrigine in patients with heart disease in clinical practice. Previous studies had suggested that bupropion, which blocked IKr ion channels, can cause QT interval prolongation when used in excess (38, 39). According to an animal study, administering therapeutic doses of metformin for a lengthy period of time (7 weeks) could prolong the duration of QTc by delay cardiac repolarization (40). There were no studies on the QT interval prolongaton brought on by letrozole or amlodipine. Using data mining techniques, the current study rapidly identified cases of drug-induced QT interval prolongation and used ROR to quantify their risk signals. Larger studies may still be required to further confirm the ADE of QT interval prolongation for medications such as letrozole, amlodipine, metformin, bupropion, and others that are not indicated for QT interval prolongation in their inserts.

Among the top 40 drugs, cisapride which was removed from the market by FDA, had the highest ROR of 312.67 (95% CI: 281.35–347.47). It prolonged the QT interval and causes serious arrhythmias by blocking potassium ion channels regulated by hERG. It is known to all that the hERG has drawn a lot of interest over the past few decades. The estimated hERG blockade and the increase in QT interval for 5 Ikr-blocking medications (haloperidol, olanzapine, risperidone, thioridazine, and ziprasidone) were found to be significantly correlated in a study involving 14 antipsychotics (41). Furthermore, genetic variations in CYP and P-glycoprotein, may impact an individual's susceptibility of drug-induced QT interval prolongation. Methadone users who had weak CYP2B6 metabolizers had been found to have an increased risk of QT interval prolongation (42). A higher risk of QT interval prolongation caused by antimicrobial drugs, with the exception of quinolones, was associated with variations of CYP3A4 and CYP2C9 (43). Variants in the CYP2D6 gene may impact exposure and result in the development of AEs associated with aripiprazole, haloperidol, and risperidone, whereas variations in the CYP3A4 gene may impact quetiapine (44). Compared to homozygous C allele carriers, risperidone users with a T allele of the C3435T polymorphism had a significantly longer QT interval (45). Therefore, it's critical to understand specific genetic backgrounds in order to lower the incidence drug-induced QT interval prolongation.

According to reports, the Weibull parameter can be utilized to forecast the period before an ADE happens and can offer helpful information for patient pharmacological management in clinical practice (16). Even though we meticulously adhered to the FDA's suggested TTO calculation, we discovered some seemingly aberrant TTO information that calls for caution, such as high median TTO values for cisapride and clozapine. Overall, most drugs that caused QT interval prolongation had early failure types, indicating that the majority of ADEs related to drug-induced QT interval prolongation occurred early in the prescribing period and then decreased over time. Early in the course of treatment, the emergence of QT interval prolongation should be closely monitored. Once QT interval prolongation is identified in a patient, the drug regimen can be changed or supportive measures can be implemented to help manage symptoms and prevent the onset of serious adverse events.

Mild QT interval prolongation is frequently asymptomatic, making it difficult for patients and physicians to recognize at first. However, when a QT interval is longer than 500 ms, there is an increased risk of cardiac events (46). Since our study, which was based on the FAERS database, was unable to gather information on whether or not patients had clinical symptoms when QT interval prolongation occurs, we simply went on to discuss the clinical outcomes which may cause by QT interval prolongation. According to the present study, 25.30% of the patients died or experienced life-threatening conditions, suggesting that the extent to which drug-induced QT interval prolongation jeopardizes patients should be of great concern. The greatest percentage of deaths or life-threatening conditions occurred in patients using metformin, which may have anything to do with the individuals' own diabetes. This was due to research suggesting that those with diabetes had a higher risk of sudden cardiac death compared to those without the disease (47).

## 5 Limitations

The real-world data mining strategy we used in our study based on the FAERS database has some advantages, several restrictions must be taken into account. These restrictions are as follows:

Firstly, FAERS is an open ADE reporting system that is widely used by the population, so it is difficult to avoid the situation of inaccurate, incomplete, and delayed reporting. Since voluntary reporting isn't restricted to healthcare professionals, ADEs may be reported voluntarily by non-healthcare professionals as well. Unfortunately, non-healthcare professionals are not very knowledgeable about medicine and are not likely to draw the right judgment**s**. These could result in inevitable bias. Secondly, reports of patients using more than one prescription raise the possibility of erroneous correlations between target drugs and target events. The utilization of disproportionality analysis to establish a definitive causal relationship between target drugs and target events was limited, as it yielded only statistical associations. These can lead to possible false positive signals in the results of the study. However, these are intrinsic limitations of spontaneous reporting databases for pharmacovigilance studies. Thirdly, the FAERS database only contains cases of ADEs, but the total number of patients taking medication is unknown. Therefore, the incidence of drug-induced QT interval prolongation was not available. Furthermore, the number of reported cases of QT interval prolongation depends on both the tendency to prolong the QT interval and the number of patients treated. Consequently, the study's findings only provide a general overview of QT interval prolongation occurrences that have been recorded in the FAERS database. Fourthly, previous research indicates that the QT interval is significantly higher in the early morning compared to the evening (48), and that the dosage of the drug has an impact on this as well (39). Owing to the restricted data contained in the FAERS database, we were unable to conduct more in-depth analysis of these factors that influence the QT interval. So we need to be cautious in the interpretation of data mining results of our study and make comprehensive judgments in combination with evidence-based medical evidence.

## 6 Conclusion

Our study offered a list of drugs that frequently caused QT interval prolongation based on the FAERS system, along with a description of some risk profiles for QT interval prolongation brought on by these drugs. When prescribing these drugs in clinical practice, we should closely monitor the occurrence of ADE for QT interval prolongation.

## Data Availability

The original contributions presented in the study are included in the article/**Supplementary Materials**, further inquiries can be directed to the corresponding author.
