# Supplementary material for: A real-world pharmacovigilance study of drug-induced QT interval prolongation: analysis of spontaneous reports submitted to FAERS
Source: Front Cardiovasc Med. 2024 May 13;11:1363382. doi: 10.3389/fcvm.2024.1363382 (PMC11128590; doi:10.3389/fcvm.2024.1363382)
Supplement: Supplementary file 1 [file Datasheet1.docx]

# Supplementary Table 1. Two-by-two contingency table for disproportionality signal analysis

|  | Target ADEs | Other ADEs | Total |
| --- | --- | --- | --- |
| Target drugs | a | b | a+b |
| Other drugs | c | d | c+d |
| Total | a+c | b+d | n=a+b+c+d |

ADE, adverse drug events.a: the number of cases with the target ADE of the target drug; b: the number of cases with all other ADEs of the target drug; c: the number of cases with the target ADE of all other drugs; d: the number of cases with all other ADEs of all other drugs.

# Supplementary Table 2. Clinical characteristics for the top 40 drugs associated with ADE of QT interval prolongation

| SN | Drug | Cases | Gender | | |  | Age | | | |  | Clinical outcomes | | |
| --- | --- | --- | --- | --- | --- | --- | --- | --- | --- | --- | --- | --- | --- | --- |
|  |  |  | Female(%) | Male(%) | Missing(%) |  | ＜18(%) | 18≤Age＜65(%) | ≥65(%) | Missing(%) |  | DE or LT(%) | Others(%) | Missing(%) |
| 1 | Quetiapine | 1151 | 640(55.60) | 392(34.06) | 119(10.34) |  | 77(6.69) | 651(56.56) | 221(19.20) | 202(17.55) |  | 276(23.98) | 843(73.24) | 32(2.78) |
| 2 | Olanzapine | 754 | 365(48.41) | 284(37.67) | 105(13.93) |  | 46(6.10) | 376(49.87) | 149(19.76) | 183(24.27) |  | 205(27.19) | 530(70.29) | 19(2.52) |
| 3 | Citalopram | 720 | 424(58.89) | 182(25.28) | 114(15.83) |  | 43(5.97) | 327(45.42) | 184(25.56) | 166(23.06) |  | 196(27.22) | 497(69.03) | 27(3.75) |
| 4 | Nilotinib | 688 | 260(37.79) | 310(45.06) | 118(17.15) |  | 4(0.58) | 186(27.03) | 202(29.36) | 296(43.02) |  | 44(6.40) | 574(83.43) | 70(10.17) |
| 5 | Clozapine | 609 | 252(41.38) | 328(53.86) | 29(4.76) |  | 7(1.15) | 383(62.89) | 31(5.09) | 188(30.87) |  | 68(11.17) | 521(85.55) | 20(3.28) |
| 6 | Amiodarone | 540 | 269(49.81) | 211(39.07) | 60(11.11) |  | 4(0.74) | 110(20.37) | 349(64.63) | 77(14.26) |  | 159(29.44) | 366(67.78) | 15(2.78) |
| 7 | Hydroxychloroquine | 511 | 193(37.77) | 206(40.31) | 112(21.92) |  | 43(8.41) | 164(32.09) | 178(34.83) | 126(24.66) |  | 129(25.24) | 366(71.62) | 16(3.13) |
| 8 | Bupropion | 489 | 248(50.72) | 116(23.72) | 125(25.56) |  | 118(24.13) | 259(52.97) | 33(6.75) | 79(16.16) |  | 198(40.49) | 290(59.30) | 1(0.20) |
| 9 | Escitalopram | 483 | 312(64.6) | 106(21.95) | 65(13.46) |  | 25(5.18) | 193(39.96) | 125(25.88) | 140(28.99) |  | 102(21.12) | 367(75.98) | 14(2.90) |
| 10 | Azithromycin | 481 | 163(33.89) | 235(48.86) | 83(17.26) |  | 18(3.74) | 169(35.14) | 197(40.96) | 97(20.17) |  | 134(27.86) | 337(70.06) | 10(2.08) |
| 11 | Ondansetron | 430 | 197(45.81) | 183(42.56) | 50(11.63) |  | 100(23.26) | 243(56.51) | 36(8.37) | 51(11.86) |  | 144(33.49) | 280(65.12) | 6(1.40) |
| 12 | Cisapride | 413 | 254(61.50) | 130(31.48) | 29(7.02) |  | 62(15.01) | 175(42.37) | 59(14.29) | 117(28.33) |  | 84(20.34) | 313(75.79) | 16(3.87) |
| 13 | Loperamide | 397 | 135(34.01) | 201(50.63) | 61(15.37) |  | 5(1.26) | 307(77.33) | 7(1.76) | 78(19.65) |  | 195(49.12) | 202(50.88) | 0(0.00) |
| 14 | Venlafaxine | 384 | 234(60.94) | 109(28.39) | 41(10.68) |  | 5(1.30) | 242(63.02) | 73(19.01) | 64(16.67) |  | 147(38.28) | 229(59.64) | 8(2.08) |
| 15 | Methadone | 374 | 115(30.75) | 218(58.29) | 41(10.96) |  | 25(6.68) | 244(65.24) | 20(5.35) | 85(22.73) |  | 123(32.89) | 237(63.37) | 14(3.74) |
| 16 | Dofetilide | 359 | 107(29.81) | 199(55.43) | 53(14.76) |  | 0(0.00) | 92(25.63) | 154(42.90) | 113(31.48) |  | 41(11.42) | 263(73.26) | 55(15.32) |
| 17 | Furosemide | 350 | 227(64.86) | 68(19.43) | 55(15.71) |  | 8(2.29) | 86(24.57) | 179(51.14) | 77(22.00) |  | 90(25.71) | 255(72.86) | 5(1.43) |
| 18 | Sertraline | 342 | 205(59.94) | 87(25.44) | 50(14.62) |  | 42(12.28) | 148(43.27) | 84(24.56) | 68(19.88) |  | 75(21.93) | 261(76.32) | 6(1.75) |
| 19 | Fingolimod | 341 | 245(71.85) | 82(24.05) | 14(4.11) |  | 1(0.29) | 263(77.13) | 16(4.69) | 61(17.89) |  | 9(2.64) | 310(90.91) | 22(6.45) |
| 20 | Aripiprazole | 339 | 163(48.08) | 103(30.38) | 73(21.53) |  | 22(6.49) | 178(52.51) | 25(7.37) | 114(33.63) |  | 85(25.07) | 249(73.45) | 5(1.47) |
| 21 | Risperidone | 317 | 130(41.01) | 103(32.49) | 84(26.50) |  | 34(10.73) | 109(34.38) | 78(24.61) | 96(30.28) |  | 73(23.03) | 234(73.82) | 10(3.15) |
| 22 | Fluoxetine | 308 | 211(68.51) | 74(24.03) | 23(7.47) |  | 41(13.31) | 171(55.52) | 56(18.18) | 40(12.99) |  | 119(38.64) | 185(60.06) | 4(1.30) |
| 23 | Ziprasidone | 273 | 135(49.45) | 85(31.14) | 53(19.41) |  | 18(6.59) | 129(47.25) | 29(10.62) | 97(35.53) |  | 56(20.51) | 181(66.30) | 36(13.19) |
| 24 | Moxifloxacin | 273 | 127(46.52) | 90(32.97) | 56(20.51) |  | 1(0.37) | 89(32.60) | 87(31.87) | 96(35.16) |  | 93(34.07) | 169(61.9) | 11(4.03) |
| 25 | Donepezil | 269 | 175(65.06) | 55(20.45) | 39(14.50) |  | 0(0.00) | 6(2.23) | 207(76.95) | 56(20.82) |  | 35(13.01) | 221(82.16) | 13(4.83) |
| 26 | Mirtazapine | 255 | 157(61.57) | 78(30.59) | 20(7.84) |  | 2(0.78) | 133(52.16) | 83(32.55) | 37(14.51) |  | 77(30.20) | 164(64.31) | 14(5.49) |
| 27 | Haloperidol | 253 | 97(38.34) | 104(41.11) | 52(20.55) |  | 9(3.56) | 111(43.87) | 54(21.34) | 79(31.23) |  | 67(26.48) | 174(68.77) | 12(4.74) |
| 28 | Ribociclib | 246 | 201(81.71) | 0(0.00) | 45(18.29) |  | 1(0.41) | 57(23.17) | 76(30.89) | 112(45.53) |  | 9(3.66) | 232(94.31) | 5(2.03) |
| 29 | Levofloxacin | 246 | 103(41.87) | 100(40.65) | 43(17.48) |  | 2(0.81) | 80(32.52) | 103(41.87) | 61(24.80) |  | 86(34.96) | 155(63.01) | 5(2.03) |
| 30 | Paroxetine | 230 | 150(65.22) | 55(23.91) | 25(10.87) |  | 5(2.17) | 110(47.83) | 55(23.91) | 60(26.09) |  | 53(23.04) | 169(73.48) | 8(3.48) |
| 31 | Amlodipine | 227 | 92(40.53) | 91(40.09) | 44(19.38) |  | 14(6.17) | 97(42.73) | 40(17.62) | 76(33.48) |  | 74(32.60) | 153(67.40) | 0(0.00) |
| 32 | Ciprofloxacin | 210 | 102(48.57) | 91(43.33) | 17(8.10) |  | 16(7.62) | 67(31.90) | 102(48.57) | 25(11.90) |  | 69(32.86) | 131(62.38) | 10(4.76) |
| 33 | Omeprazole | 208 | 121(58.17) | 79(37.98) | 8(3.85) |  | 23(11.06) | 97(46.63) | 69(33.17) | 19(9.13) |  | 44(21.15) | 161(77.40) | 3(1.44) |
| 34 | Bedaquiline | 205 | 68(33.17) | 117(57.07) | 20(9.76) |  | 2(0.98) | 162(79.02) | 14(6.83) | 27(13.17) |  | 73(35.61) | 130(63.41) | 2(0.98) |
| 35 | Clarithromycin | 194 | 94(48.45) | 83(42.78) | 17(8.76) |  | 3(1.55) | 77(39.69) | 89(45.88) | 25(12.89) |  | 62(31.96) | 131(67.53) | 1(0.52) |
| 36 | Letrozole | 193 | 179(92.75) | 1(0.52) | 13(6.74) |  | 0(0.00) | 61(31.61) | 63(32.64) | 69(35.75) |  | 6(3.11) | 186(96.37) | 1(0.52) |
| 37 | Duloxetine | 175 | 108(61.71) | 54(30.86) | 13(7.43) |  | 5(2.86) | 79(45.14) | 42(24.00) | 49(28.00) |  | 38(21.71) | 130(74.29) | 7(4.00) |
| 38 | Lamotrigine | 159 | 101(63.52) | 36(22.64) | 22(13.84) |  | 13(8.18) | 112(70.44) | 10(6.29) | 24(15.09) |  | 55(34.59) | 104(65.41) | 0(0.00) |
| 39 | Fluconazole | 156 | 90(57.69) | 60(38.46) | 6(3.85) |  | 9(5.77) | 93(59.62) | 33(21.15) | 21(13.46) |  | 46(29.49) | 109(69.87) | 1(0.64) |
| 40 | Metformin | 155 | 87(56.13) | 52(33.55) | 16(10.32) |  | 0(0.00) | 87(56.13) | 37(23.87) | 31(20.00) |  | 82(52.90) | 73(47.10) | 0(0.00) |
| Total | | 14707 | 7536(51.24) | 5158(35.07) | 2013(13.69) |  | 853(5.80) | 6723(45.71) | 3649(24.81) | 3482(23.68) |  | 3721(25.30) | 10482(71.27) | 504(3.43) |

ADE, Adverse drug event; DE, death; LT, life-threatening; SN, serial number
